# *In Vitro* and *In Vivo* Evaluation of the Skincare Bioactivity of β-1,3;1,6-Glucans-Rich *Ganoderma lucidum* Polysaccharides

**DOI:** 10.3390/molecules31101740

**Published:** 2026-05-20

**Authors:** Cheng-Fu Huang, Jia-Feng Chang, Hui-Shan Yang, Chih-Ping Hsu, Chih-Cheng Lin

**Affiliations:** 1 Department of Biotechnology and Pharmaceutical Technology, Yuanpei University of Medical Technology, Hsinchu 300, Taiwan; jien0989176748@yahoo.com.tw; 2 Department of Nursing, Yuanpei University of Medical Technology, Hsinchu 300, Taiwan; cjf6699@gmail.com; 3 Division of Nephrology, Department of Internal Medicine, Taoyuan Branch of Taipei Veterans General Hospital, Taoyuan 330, Taiwan; 4 Department of Chemical Engineering and Biotechnology, National Taipei University of Technology, Taipei 10608, Taiwan; gigishanbad@gmail.com; 5 Department of Medical Laboratory Science and Biotechnology, Yuanpei University of Medical Technology, Hsinchu 300, Taiwan; hsucp@mail.ypu.edu.tw

**Keywords:** active polysaccharide, skincare, *Ganoderma lucidum*, β-glucan, wound healing

## Abstract

Extracted from *Ganoderma lucidum* mycelium, the developed β-1,3;1,6-glucan rich polysaccharides have the potential to be used during the industrial production of health food products due to their inhibition of metabolic syndrome, immunomodulatory and antitumor activities and other health benefits. *Ganoderma* active polysaccharides (GAP) have also been found to promote skin health, particularly due to their antioxidant and anti-aging properties. The present study investigates the skin-protective properties of polysaccharides purified from *Ganoderma* mycelium cultivated using stress-tolerance technology and a fully plant-based medium. The effects of the GAP are investigated in both *in vitro* and human studies. The results of the study indicate that the developed GAP effectively inhibit 32.4% of tyrosinase activity and 30.6% of melanin production in B16F10 cells. Furthermore, in scratch assays using NIH 3T3 cells, these GAP also promote cell migration and wound healing. In human studies, GAP demonstrated no potential for skin irritation while effectively reducing skin wrinkles, enhancing skin brightness, diminishing erythema, and increasing epidermal hydration. In hot-flux patch-induced erythema experiments, these GAP were found to be capable of alleviating erythema severity by up to 48%. The present study demonstrates that GAP, which can be produced industrially using innovative technologies and is rich in highly water-soluble β-1,3;1,6-glucan with a triple-helix structure, holds potential for application in the skincare industry.

## 1. Introduction

The term “biocosmetics” is used to denote cosmetics that are derived from biological sources, including but not limited to plants, animals, or microorganisms. The replacement of synthetic chemicals with natural ingredients has been demonstrated to minimize skin irritation and allergic reactions, while concomitantly promoting environmental sustainability [1]. A significant number of studies have been conducted that present the findings of research and surveys exploring the potential of fungi or mushrooms as ingredients in cosmeceuticals [2,3]. Fungi such as *Ganoderma* sp. are considered to be a traditional source of naturally occurring bioactive compounds [4]. These can be used in a variety of ways in relation to cosmetics [2]. Such benefits include exfoliation of dead skin cells, the mitigation of inflammation, and the acceleration of skin cell renewal. Furthermore, the aforementioned substances have been demonstrated to enhance the elasticity of the skin and promote its whitening [5,6,7].

*Ganoderma lucidum* (Curtis) P. Karst. (*G. lucidum*, Lingzhi), a species of mushroom, has been demonstrated to possess bioactivities that are conducive to dermal health, and it has been shown to ameliorate dermatological conditions [8]. It has been reported that polysaccharides constitute the predominant functional metabolites of *Ganoderma*, which have been shown to possess antioxidant properties [9,10,11,12]. Furthermore, *G. lucidum* has been demonstrated to have significant potential in skincare cosmetics for the treatment of skin hyperpigmentation [13,14]. *G. lucidum* polysaccharide has a significant positive effect on the viability and migratory ability of fibroblasts, as well as wound healing rates [15]. The study suggests that polysaccharide has the potential to promote skin wound healing by activating the Wnt/β-catenin signaling pathway and up-regulating TGF-β1. Extracted polysaccharides from *G. lucidum* have been employed to promote the healing of skin wounds and to mitigate post-burn infection. Furthermore, *Ganoderma* extracts have been incorporated into skincare products due to their recognized efficacy in mitigating photoaging and promoting skin lightening. The anti-inflammatory properties of *Ganoderma* have been demonstrated in models of atopic dermatitis and cutaneous sarcoidosis, suggesting its potential for use in the treatment of skin carcinoma [16]. Among mushroom extracts, β-glucans is the most important active ingredient that boost the effectiveness of cosmetic products. The *in vitro* and *in vivo* studies that have been conducted have demonstrated that β-glucans have a significant effect on skincare and wound healing [17]. It has been demonstrated that β-glucans, which are derived from mushrooms, have the capacity to promote keratinocyte migration, thereby accelerating wound healing [18]. In addition to this, they have been shown to activate dermal fibroblast differentiation to remodel the matrix. In the treatment of chronic wounds, β-glucan has been demonstrated to promote collagen synthesis through multiple pathways and to regulate the ratio of type I to type III collagen, thereby helping to reduce scar formation [19].

Although polysaccharides can be extracted from *G. lucidum* directly, they can also be produced through biotechnological cultivation to yield extracellular polysaccharides. Exopolysaccharides produced by fungal mycelium during submerged fermentation offer significant advantages over those extracted from mycelium or fruiting bodies. These exopolysaccharides have been demonstrated to facilitate enhanced yields within reduced production cycles, whilst concurrently exhibiting reduced susceptibility to contamination [20]. The *G. lucidum* active polysaccharides (GAP) were processed using metabolic synthesis technology and contained about 95.9% carbohydrates and 51.7 water-soluble polysaccharides in our previous study [21]. These polysaccharides, which have been found to exhibit inhibitory effects on metabolic syndrome, anti-inflammatory properties, and antitumor activity, were found to originate primarily from β-1,3;1,6-glucan, present at a concentration as high as 73% [21]. Most studies on the effects of fungal extracts produced by biotechnology on the skin have been conducted through cell and animal experiments. However, researchers have conducted few actual human trials or clinical research. This study uses polysaccharides rich in β-1,3;1,6-glucan extracted from cultured *G. lucidum* mycelium to evaluate their *in vitro* activities related to skin health and to investigate the efficacy of this substance when applied to human skin.

## 2. Results

### 2.1. In Vitro Study

#### 2.1.1. The Effect of GAP on DPPH Scavenging Ability, Inhibition of Tyrosinase Activity and Melanin Formation

*G. lucidum* mycelium cultivated using plant-based media and stress-resistant techniques, combined with patented purification technology, yields a crude extract of GAP at a concentration of 16 mg/mL. As shown in Table 1, the DPPH radical scavenging assay indicates that the GAP extract exhibits only a 16.87% inhibition rate against DPPH radicals. However, upon dilution to half its original concentration, GAP exhibits a complete loss of scavenging capacity, thereby suggesting that GAP possesses relatively low antioxidant activity in purely chemical reactions. These lower results differ from some other findings [9,11] and may be related to differences in the molecular size and structure of the polysaccharides obtained through the extraction and purification methods [12].

An eight-fold dilution of GAP (2 mg/mL) exhibited no significant toxicity to cell viability in B16-F10 cells. The findings, as presented in Table 1, indicate that the GAP compound demonstrated a substantial inhibitory influence, evidenced by a 21.93% decrease in intracellular tyrosinase activity and a 30% reduction in melanin formation. These findings are consistent with those of previous studies [12,22,23,24], which suggest that polysaccharides derived from *G. lucidum* mycelium may inhibit tyrosinase activity through competitive inhibition or by altering tyrosinase conformation. The formation of melanin in B16-F10 cells was found to be reduced as a consequence of GAP’s inhibition of tyrosinase activity and its protein expression.

#### 2.1.2. Wound Healing Ability of GAP

**Figure 1:**
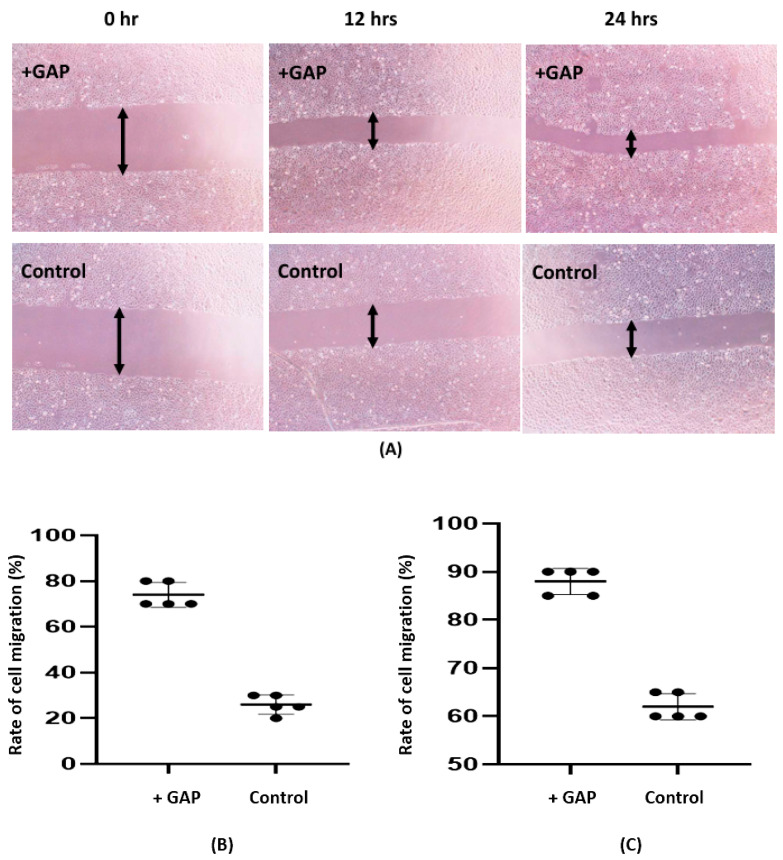
Effect of GAP on wound healing activity in the scratch assay. (**A**) Representative images of cell migration. Cell migration rates after 12 h (**B**) and 24 h (**C**) of treatment. Individual data points represent independent biological replicates (*n* = 5), and horizontal bars indicate the mean ± SD.

Given that β-glucan has been demonstrated to induce the production of growth factors that are essential for skin, as well as to promote collagen biosynthesis and maintain skin moisture and elasticity [17,18], the present study investigated the potential of GAP to regenerate damaged skin in scratch assays within NIH 3T3 cells. The tested GAP demonstrated enhanced fibroblast proliferation and/or migration capacity in scratch assays (Figure 1A). The average proliferation/migration rate of the experimental group was found to be 2.5 times higher than that of the control group at 12 h (see Figure 1B). Moreover, 90% wound healing was achieved at 24 h (see Figure 1C). The rate of migration increased by 15% over 24 h, suggesting that GAP are more effective in promoting wound healing during the initial phase.

### 2.2. In Vivo Study

#### 2.2.1. Skin Assessments of GAP

Prior to conducting human trials on the skin efficacy of GAP, Insult Patch Tests (HRIPT) were performed on leave-on cosmetic products, as outlined in the COLIPA Guidelines. The purpose of the HRIPT was to confirm the absence of irritation side effects under normal usage conditions. In a 24 h test involving ten subjects, the Mean Irritation Index was 0.1, classified as non-irritating according to the irritation-grading standard. Within the specified 48 h period, no subjects reported experiencing sensory discomfort, defined as the presence of stinging, burning, or itching sensations. These results demonstrate the excellent skin compatibility of GAP. A total of thirty subjects applied GAP to the butterfly area of their faces for a period of 28 consecutive days. An analysis of the skin measurements presented in Table 2 indicates that the GAP treatment led to a significant reduction in skin wrinkles by 3.6%, an enhancement in skin brightness by 10.2%, a decrease in erythema by 3.0%, and an increase in epidermal moisture content by 7.5%. The results obtained in this study were consistent with those reported in other studies [4,5,7,17], indicating statistically significant differences, particularly with regard to skin-brightening and moisture-retaining capabilities.

#### 2.2.2. Erythema-Reducing Efficacy of GAP

The manifestation of skin erythema can be attributed to a multitude of factors. The most prevalent primary causes encompass prolonged sun exposure and inflammatory conditions associated with skin diseases, manifesting as signs of hyperpigmentation and warmth [25]. With regard to the efficacy of GAP in alleviating erythema, two test areas were established on the inner arms of ten subjects: an experimental area where GAP was applied after hot-flux patch stimulation, and a control area where no GAP was applied. The levels of redness were measured at 30 and 60 min after application of the product. As demonstrated in Table 3, the application of GAP resulted in a 13.91% reduction in erythema severity after 60 min, surpassing the 9.39% reduction observed in the untreated control group. In comparison with the control group, GAP demonstrated a 48% enhancement in the relief of erythema. These findings are consistent with those of other studies [5,6,17,25], indicating that the application of GAP is a highly effective treatment modality for the management of erythema arising from skin irritation.

## 3. Discussion

It is evident that β-Glucan exerts considerable biological activity and demonstrates considerable potential for application in the domain of skincare [17]. The primary benefits include immune modulation, antioxidant effects, anti-inflammatory properties, skin barrier repair, and moisturizing nourishment. It is noteworthy that β-glucan also exhibits considerable potential in the treatment of skin conditions, including the promotion of wound healing, protection against UV damage, the improvement of photoaging, and the alleviation of atopic dermatitis [17]. The present study corroborates the hypothesis that glucans with a high proportion of β-1,3;1,6-glucans, obtained from *G. lucidum* mycelium cultivated in plant-based media, have skincare and therapeutic potential in the fields of dermatology and medicine. This evidence is supported by *in vitro* cellular experimentation and *in vivo* human trials. Despite the results of low antioxidant activity presented in the research, it is understood that *Ganoderma* polysaccharides do not exhibit superior chemical free radical scavenging capacity [9,11]. Instead, the evidence suggests that they demonstrate antioxidant effects by inducing the activity of superoxide dismutase (SOD), catalase (CAT), and glutathione peroxidase (GSP) in biological organisms [10]. *Ganoderma* polysaccharides have been shown to activate antioxidant enzymes such as CAT, SOD, and by increasing the expression of antioxidant genes like NQO1 and NO-1, thereby counteracting oxidative stress [12]. Cellular experimentation has indicated that the observed dermal effects of the GAP substance in human trials, including whitening, moisturizing, reducing erythema, and promoting fibroblast proliferation, are not the result of straightforward chemical reactions. Rather, these effects are attributed to its capacity to modulate cellular signaling pathways. It is well established that β-1,3;1,6-glucans typically exert potent signaling and regulatory effects, primarily due to their stable triple-helix structure [26]. The long-chain β-1,3-glucan backbone forms the core helix, while β-1,6 glycosidic bonds introduce branching structures, which enhance solubility, hydration and influence receptor binding in the pathways they regulate.

Skin wound healing is a complex process, generally divided into three stages: inflammation, proliferation, and tissue remodeling. Multiple studies have shown that β-glucan has potential in promoting wound healing. A plethora of studies have devised various strategies to enhance wound healing and limit scar formation by regulating the wound healing process. In comparison with synthetic polymers, natural polysaccharides are considered to be more appropriate for use as wound healing components due to their biodegradability, biocompatibility, and low toxicity [27]. The present results of the developed GAP produced from *G. lucidum* through biotechnology and the polysaccharides traditionally extracted from *G. amboinense* or other sources are comparable [28,29], demonstrating highly effective cellular wound healing effects. Consequently, the GAP possess considerable potential as novel resources for the development of wound-healing cosmetics.

Fungal polysaccharides offer distinct advantages that are in alignment with the current trends in the cosmetic industry towards sustainable development. The utilization of organic materials ensures a sustainable alternative to synthetic ingredients, catering to the growing demand from environmentally conscious consumers. This study signifies the first application of innovative metabolic synthesis techniques in cellular and *in vivo* models to evaluate the benefits of active polysaccharides from *G. lucidum* on the skin. The results clearly demonstrate that these GAP, which are rich in β-1,3;1,6-glucan in our previous study [21], can be regarded as an optimal source of cosmetic ingredients and exhibit broad potential for use in skin therapy. In addition, this water-soluble GAP is both environmentally sustainable and continuously producible, and is generally easier to formulate into skincare products because it emulsifies readily, forming stable lotions or creams suitable for developing lightweight formulations. Furthermore, water-soluble ingredients are generally more readily absorbed by the skin, as the skin’s natural barrier permits water molecules to pass through more easily, rendering them suitable for use in the development of moisturizing products.

## 4. Materials and Methods

### 4.1. Sample Preparation

The mycelium employed for the purpose of inoculum preparation and polysaccharide production is the *G. lucidum* strain CCRC 36792 (Biological Resource Center and Research Institute, Hsinchu, Taiwan), which has been optimized through the Strain Optimization System (TWN Patent M640778). Fermentation was conducted using plant-based media and incorporating stress management operations using a previously described method [21]. Extracellular polysaccharides were produced through metabolic engineering and the fermentation broth was subjected to a process of concentration via a purification system (TWN Patent M646622) to define *G. lucidum* active polysaccharides (GAP). The GAP employed in this study was supplied by DISAM Biotechnology Co., Ltd. (Taipei, Taiwan).

### 4.2. DPPH Scavenging Ability

The preparation of the reaction reagent involves the dissolution of 7.87 mg of DPPH in 10 mL of DMSO. Subsequently, GAP is added to the DPPH solution, and the reaction is allowed to proceed at room temperature for a duration of 60 min. Subsequent to the reaction, the spectrophotometer should be utilized to measure the extinction coefficient at a wavelength of 517 nm. The DPPH scavenging capacity can be calculated by determining the percentage of decrease in the absorbance.

### 4.3. Inhibition of Tyrosinase Activity and Melanin Formation

The murine melanoma B16-F10 cell (BCRC 60031) cell line was cultured in Dulbecco’s Modified Eagle Medium (DMEM), supplemented with 10% fetal bovine serum (FBS). All cells were incubated in a humidified incubator with 5% CO_2_ at 37 °C. Cells were regularly monitored and maintained at sub-confluent levels to ensure optimal growth conditions. The medium was refreshed every 48 h, and cells were passaged upon reaching 80–90% confluence and 0.25% trypsin-EDTA solution was used to detach the cells.

The B16-F10 cells were introduced to a 96-well cell culture plate, with 100 μL of complete culture medium, at an initial density of 1 × 10^4^ cells/well. The plate was subsequently placed in a 37 °C incubator containing 5% CO_2_, for a 24 h incubation period. The following day, GAP should be added and the incubation continued for a period of 72 h. Following the incubation period, 50 μL of MTT solution (1 mg/mL) should be added. The samples are then subjected to an incubation process at a temperature of 37 °C for a duration of two hours. Following this, 100 μL of DMSO is added to the samples. Following dissolution, the measurement of the sample’s optical density at a wavelength of 570 nm is to be conducted using an ELISA reader (Tecan Group Ltd., Männedorf, Switzerland), thus enabling the calculation of the cell viability.

**Figure 2:**
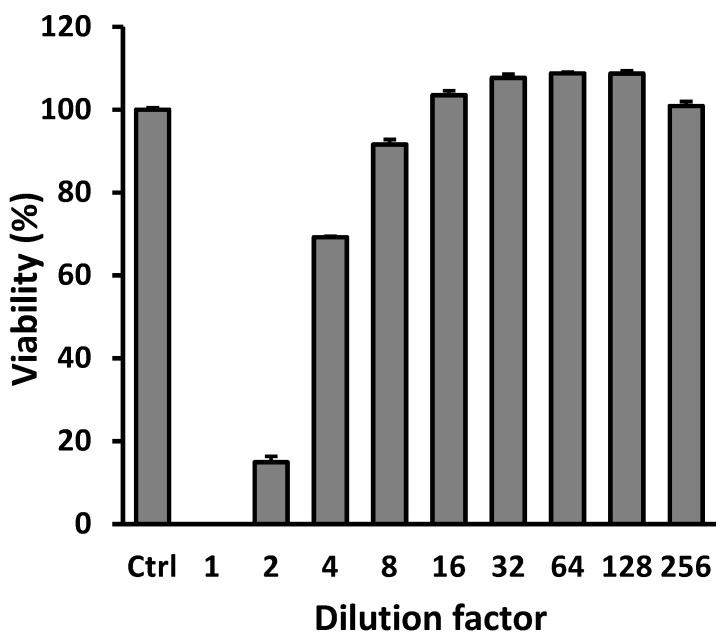
Cytotoxicity of GAP against B16-F10 cells.

The GAP stock solution is characterized by a concentration of 16 mg/mL. In order to ascertain whether GAP exerts a cytotoxic effect on B16-F10 cells, the experiment involved the addition of GAP to B16-F10 cells at various dilution factors. As demonstrated in Figure 2, GAP demonstrates no evidence of toxicity at dilutions of 8-fold or higher, i.e., at concentrations equal to or lower than 2 mg/mL.

Subsequently, 2.5 × 10^4^ cells of the B16-F10 strain were seeded into 6-well plates, which had been pre-filled with 2 mL of culture medium. Then, the plates were subjected to an incubation period at 37 °C within a 5% CO_2_ incubator for a duration of 24 h. Subsequent to the incorporation of GAP, the incubation period should be maintained for a duration of 72 h. Subsequently, the cells should be dissolved using 20 mM 0.1% Triton X-100, after which the mixture should be subjected to centrifugation. A portion of the resulting supernatant should then be collected for the purpose of protein quantification. The supernantant should be added to the L-DOPA solution, which is then to be incubated at 37 °C for a period of 10 min. Subsequently, the degree of absorption at 475 nm should be measured. The rate of tyrosinase activity inhibition can be determined by calculating 1-(OD475 of the test sample/OD475 of the control) multiplied by 100. As described above, the lysed cells were treated with 20% and 10% TCA, respectively, to precipitate proteins. Following the washing of the samples with diethyl ether, they were left to dry in air. Thereafter, they were dissolved in 0.85 M KOH, and the absorbance at 440 nm were measured. The inhibition rate of melanin production can be calculated by determining the difference between the optical density (OD) of the sample and the control at a wavelength of 440 nm, and then dividing this by 100 [30].

### 4.4. Wound Healing Scratch Assay

The migration of NIH 3T3 fibroblasts was assessed using the wound healing scratch assay. The cells were seeded in 24-well tissue culture dishes for 24 h at 37 °C, at a concentration of 7.6 × 10^4^ cells/mL, and cultured in 1 mL of medium containing 10% fetal bovine serum until a nearly confluent cell monolayer was achieved. A sterile pipette tip is then utilized to create a linear scratch on the monolayer of cells. Thereafter, 500 µL of fresh medium is introduced into the culture, along with 2 mg/mL of GAP (GAP, *in vitro*). The experiment is to be replicated three times. The cells must then be incubated at 37 °C for 21 h. Prior to and following the incubation period, three images of each well were captured using an Olympus IX70 microscope (Hachioji-shi, Japan) in order to assess cell proliferation and/or migration. The migration rates of cells were calculated based on the percentage of scar closure at 12 and 24 h [31].

### 4.5. Human Study

Thirty participants in this human study were recruited through advertisements. The inclusion criteria encompassed women within the age range of 20 to 50 years, who were not currently utilizing any medications and were not pregnant. The present study was conducted in accordance with the recommendations of the National Cheng Kung University Human Research Ethics Committee, and all participants provided written informed consent. The present study protocol was approved by the Institutional Review Board (NCKU HREC-E-113-176-2). A dose of 2 mg/cm^2^ of GAP was applied to the facial skin test area twice daily, in the morning and evening, for a period of 28 consecutive days. Participants were instructed to avoid excessive sun exposure in the test area during the study period. Skin assessments were conducted prior to and following treatment in a temperature- and humidity-controlled room (65% humidity and 26 °C) to analyze skin fine lines (Miravex Antera 3D Analyzer, Miravex Limited, Dublin, Ireland), skin tone and erythema (Mexameter MX18, Courage+Khazaka electronic GmbH, Köln, Germany), and skin moisture content (Corneometer 825, Courage+Khazaka electronic GmbH, Köln, Germany).

In the erythema-reducing efficacy test, the inner sides of the arms of 10 subjects were divided into two areas, a GAP application group and a control group, each measuring 3 × 3 cm^2^. Following the application of a 1% vanillyl butyl ether (Hot-Flux) occlusive patch to the two test areas for 30 and 60 min, respectively, the erythema-reducing effect was evaluated using a Minolta Chromameter (Konica Minolta, Tokyo, Japan).

### 4.6. Statistical Analysis

The data are presented as standard deviations (SDs) from four replicate experiments. In order to ascertain whether the observed differences between the treatment group and the control group were statistically significant, a one-way analysis of variance (ANOVA) was conducted, followed by Dunnett’s multiple comparison test. The analysis of the human trials was conducted using a paired *t*-test. A *p*-value of less than 0.05 was considered to be statistically significant.

## 5. Conclusions

The present study is the first to demonstrate *in vitro* and human clinical trials that high-concentration β-1,3;1,6-glucan, produced from *Ganoderma lucidum* mycelium using a plant-based culture medium, is highly effective in brightening skin tone, hydrating the skin, alleviating erythema, and promoting wound healing. It is widely acknowledged that the ingredient under discussion is considered suitable for use as a key component in skincare products, on account of its eco-friendly properties and high water solubility.

## 6. Patents

The *Ganoderma* mycelium cultivation system was developed using TWN Patent M640778 and purified using TWN Patent M646622.

## Figures and Tables

**Table 1:** Inhibitory activity of *Ganoderma lucidum* polysaccharides *in vitro* study.

	Inhibition % (Mean ± SD)
DPPH scavenging ability	16.9 ± 0.8
Tyrosinase activity	32.4 ± 1.6
Melanin content	30.6 ± 2.4

The GAP concentration used in the DPPH assay was 16 mg/mL, while the concentration employed in the B16-F10 cellular assay for inhibiting tyrosinase and melanin production was 2 mg/mL. Data are presented as mean ± S.D., *n* = 4.

**Table 2:** Effect of *Ganoderma lucidum* polysaccharide treatment after 28 days of *in vivo* study.

	Before	After	Difference	Significance
Wrinkle level	4.47	4.31	−0.16	*
Melanin Index	125.6	112.8	−12.8	***
Erythema level	209.0	202.8	−6.2	*
Hydration	31.57	33.93	2.36	**

A significant difference at the level of * *p* < 0.05, ** *p* < 0.01, *** *p* < 0.001.

**Table 3:** Reduction of skin erythema by *Ganoderma lucidum* polysaccharides following hot-flux patch stimulation.

	Control	Treatment
Before irradiation	9.74	9.46
After irradiation	13.74	13.73
After 30 min treatment	13.22	13.12 *
After 60 min treatment	13.45	11.82 **

A significant difference at the level of * *p* < 0.05, ** *p* < 0.01.

## Data Availability

All raw data are available upon request from the corresponding author.
